# Health Education and Its Role in the Prevention and Management of Non-communicable Diseases: A Review

**DOI:** 10.7759/cureus.92207

**Published:** 2025-09-13

**Authors:** Ahmed Rudwan, Leena Saeed, Layla M Mahir, Maab Ibrahim, Lina H Hasabelgawi, Heyam Rudwan

**Affiliations:** 1 Research, Hamad Medical Corporation, Doha, QAT; 2 General Medicine, Ministry of Public Health, Doha, QAT; 3 General Medicine, The National Ribat University, Khartoum, SDN; 4 Medicine, Ministry of Public Health, Doha, QAT; 5 Research, Sidra Medicine, Doha, QAT; 6 Internal Medicine, Hamad Medical Corporation, Doha, QAT

**Keywords:** behavior change, health education, health promotion, literacy, non-communicable diseases, prevention

## Abstract

Non-communicable diseases have become a major global health concern, largely due to factors that can be influenced by lifestyle choices. Behaviors such as unhealthy eating, physical inactivity, tobacco use, and excessive alcohol consumption contribute significantly to the rise of these conditions. Promoting health education plays an important role in addressing this challenge by supporting better understanding, encouraging healthier behaviors, and facilitating early recognition of symptoms. A systematic search of PubMed and Web of Science was conducted for studies addressing health education interventions, including formal education, peer-led programs, digital tools, and media campaigns. Both quantitative and qualitative outcomes were considered.

A range of strategies, from formal education systems to digital tools and media campaigns, have been used to tackle these issues. Despite these efforts, obstacles remain, including gaps in health infrastructure, cultural norms, and limited access to education. Improving coordination across sectors, ensuring stable resources, and designing approaches that reflect local needs can help ease the burden of these diseases. Moving forward, adapting solutions to different contexts and expanding access to education will be key to improving outcomes.

## Introduction and background

Non-communicable diseases (NCDs) are chronic conditions not caused by acute infections, characterized by long duration and the need for continuous care. Chronic diseases, often known as NCDs, are long-lasting conditions that develop gradually. NCDs typically arise from an interaction of genetic predisposition, behavioral patterns, physiological changes, and environmental exposures [1]. Nearly 75% of all deaths globally, or 43 million deaths annually, are caused by NCDs, as stated by the World Health Organization (WHO) [2]. The top four NCD killers per year are diabetes (over 2 million deaths), respiratory illnesses (4 million deaths), cancer (10 million deaths), and cardiovascular diseases (19 million deaths) [2]. Diabetes, cancer, respiratory disorders, and cardiovascular diseases account for almost 82% of early NCD deaths. Air pollution, poor eating habits, alcohol misuse, sedentary lifestyles, and tobacco use are the primary causes of chronic diseases. These factors significantly increase the risk of dying from NCDs. The global response to NCDs must include palliative care, early screening, diagnosis, and treatment [2].

Health education is an ongoing, changing, multifaceted, and planned educational process that occurs in a variety of settings and across the lifespan through an equitable and mediated partnership between a client and a healthcare provider. Its goal is to help initiate and support behavioral changes in people related to their lifestyle, which improve their health. Within a positive health perspective, health education considers both internal and environmental variables that influence the health of individuals and communities. Consequently, their knowledge, attitudes, and beliefs regarding their health-related needs and behaviors could all improve [3]. Health education is critical for avoiding NCDs because it provides individuals with the knowledge and the skills they need to lead healthier lifestyles and make informed health decisions. In addition to its importance in combating the worldwide NCD pandemic, it raises the knowledge of risk factors, encourages early identification, and creates supportive settings.

For this narrative review, literature was identified through searches of PubMed and Google Scholar using keywords such as “health education,” “health literacy,” “non-communicable diseases,” “prevention,” and “behavior change.” Articles published in English between 2000 and 2025 were considered. Peer-reviewed studies, systematic reviews, and relevant reports focusing on health education strategies, interventions, or policies for NCD prevention and control were included. Studies not available in full text, non-English publications, conference abstracts, and unrelated articles were excluded. The final selection was guided by relevance, methodological quality, and contribution to the review objectives.

This review aims to outline the global impact and burden of NCDs, assess the critical role of health education in their prevention and management, and examine various educational approaches, including formal, community-based, and communication strategies. It also identifies key barriers to effective health education, highlights evidence-based methods to strengthen such programs, and synthesizes current findings while identifying gaps in education related to NCD risk.

## Review

Role of health education in NCD prevention and control

Formal Education and Foundational Knowledge

Role of general education: Health literacy refers to the ability to access, analyze, evaluate, and use health information in order to make educated health decisions [4]. It comprises reading, interpreting, and using health-related information to enhance well-being [4]. Today, health literacy is seen as a potential mechanism through which the relationship between education and health can be explored, understood, and explained [5]. Accessing trustworthy health information, comprehending medical jargon, evaluating health information, and using it successfully are all important components of health literacy. Research shows that limited health literacy is linked to worse health outcomes and less effective use of healthcare services [6,7] and elevated medical expenditures [8]. Health literacy enhances communication between patients and healthcare professionals, empowers people, lowers health inequities, and improves health outcomes. Programs for health education are essential for fostering health literacy because they provide people with the information, abilities, and self-assurance they need to manage their health properly [9].

Numeracy is the capacity to comprehend and apply numerical data to make well-informed health decisions and is a key component of health education. It requires skills such as controlling drug dosages, comprehending risk probabilities, and analyzing graphs [10]. Informed health decisions, efficient communication with healthcare professionals, self-management of health, risk assessment and management, and preventing health mistakes all depend on health numeracy [11]. Low numeracy, communication gaps, and information design are obstacles; however, targeted instruction, transparent communication, easily available materials, and an emphasis on real-world application are crucial for enhancing health numeracy [11]. Healthcare professionals should explain numerical data using visual aids, avoid technical jargon, and speak plainly. Emphasizing real-life applications makes health numeracy training more engaging and ensures better retention of knowledge.

Biology lays the groundwork for understanding the human body, preventing disease, encouraging healthy lifestyle choices, comprehending environmental effects, and influencing medical procedures, making it crucial to health education. It offers the scientific foundation for medical treatments and public health initiatives, explains how body parts interact, prevents illnesses, and encourages healthy lives. Athanasiou [12] outlined the development and evaluation of a course integrating biology and environmental health education to enhance the scientific literacy of future teachers. The course addressed chronic diseases, such as cancer, diabetes, and cardiovascular disease, by exploring their links to lifestyle and environmental factors. Topics included nutrition, smoking-related risks, genetic diseases, and reproductive health. An evaluation with 153 students showed high engagement and positive learning outcomes. The study highlighted the importance of incorporating environmental health within biology and health education to prepare educators for teaching chronic disease prevention effectively. 

School curricula and long-term impacts on lifestyle: Children and adolescents are ideal targets for behavioral interventions due to their adaptability and receptiveness to adopting healthy habits. Early adoption of healthy behaviors is more successful, and programs and policies that support them are required. Schools serve a broad population and provide health-related instructional activities, making them perfect places to model, encourage, and reinforce healthy habits in children and teenagers. By teaching and instilling healthy behaviors in pupils, schools serve as centers for health. A review by Singh et al. [13] reported that school policies are more successful in improving unhealthy dietary habits, reducing tobacco use, increasing physical activity, and lowering inflammatory markers compared to their impact on body measurements, overweight or obesity rates, and alcohol consumption. Across 103 different outcomes assessed, nearly half (46%) showed significant positive changes linked to school policy interventions. The authors emphasized that schools have the potential to play a key role in promoting healthier behaviors related to NCD risk factors but highlighted the need for further studies that track long-term effectiveness and sustainability of these interventions.

Case examples of education from different countries: Several case examples illustrate the impact of education on health outcomes across different countries. In India, Pati et al. [14] emphasized the need for a comprehensive and integrated approach to teaching in medical education, particularly to help students understand the relevance of pre-clinical and para-clinical learning in community medicine. Two key strategies, horizontal and vertical integration, are essential when educating students about NCDs and their risk factors. Horizontal integration involves teaching related subjects (such as physiology, pathology, and pharmacology) simultaneously, allowing students to grasp how different disciplines interconnect. Vertical integration links basic sciences with clinical subjects across different stages of the medical curriculum, enabling students to apply foundational knowledge in real-life clinical and community health settings. This integrated model helps students gain a deeper understanding of NCD management, which often affects multiple organ systems. The revised Medical Council of India (MCI) curriculum, now known as the National Medical Commission (NMC) curriculum, supports this integrated approach by promoting competency-based learning, early clinical exposure, and interdisciplinary teaching.

In Jordan, a study involving 178 undergraduate nursing students assessed the impact of a three-week interactive educational intervention on chronic NCDs, including diabetes, hypertension, and cancer. Using a computer-based questionnaire, researchers evaluated students’ knowledge and attitudes before and after the sessions. The findings showed a significant initial improvement, particularly among female students, in both knowledge and health-promoting attitudes. However, these improvements were not sustained over time, highlighting the need for continued reinforcement. The study concluded that promoting healthy behaviors among young adults requires the integration of innovative, ongoing health education strategies into university curricula [15].

Across the Gulf and Arab countries, lifestyle intervention programs targeting adults, such as changes in workplace nutrition, fitness promotion, and community-based health awareness, have shown positive outcomes for reducing chronic disease risk [16]. Community-centered behavior modification and education programs are effective, as demonstrated in Lebanon, where mobile health (mHealth) messaging programs for managing diabetes and hypertension showed high user satisfaction and reported behavioral improvements [17].

Recent systematic reviews and meta-analyses have highlighted the significant role of eHealth and mHealth interventions in managing NCDs following the COVID-19 pandemic. A comprehensive meta-analysis encompassing 73 randomized controlled trials with over 18,000 participants demonstrated that digital health interventions significantly improved physical activity levels, reduced sedentary behavior, enhanced dietary habits, and promoted better sleep quality. Specifically, interventions led to an increase of 1329 steps per day, 55 minutes per week of moderate-to-vigorous physical activity, a decrease of 426 minutes per week in sedentary behavior, and improvements in fruit and vegetable consumption and energy intake (p<0.05) [18]. These findings underscore the potential of digital health tools to complement traditional community- and school-based interventions, especially in the post-COVID era where remote health solutions have become increasingly important. Integrating digital health interventions into existing health education strategies can enhance accessibility, continuity of care, and patient engagement, thereby contributing to more effective NCD prevention and management.

The United Arab Emirates (UAE)’s physical and health education reform is a structural intervention aimed at preventing NCDs by embedding health promotion into the school system, including curriculum changes, increased physical activity, and health education. By changing the school environment and teaching approach, this reform supports large-scale, sustainable behavior change and promotes long-term public health [19].

Research in Indonesia investigated the effectiveness of a peer-educator program to encourage healthy lifestyle choices among teenagers, using a 20-week 'experiential learning' technique to develop the 'POSBINDU' (Integrated Counseling Post) program. Ten general practitioners (GPs) were trained as part of the program, fifty peer-educators were recruited and observed in high schools, health lifestyle modules were created, and the program was implemented. Perceptions and knowledge of NCDs significantly improved for both students and general practitioners. The study concluded that the program may be enhanced with the right interventions to encourage students to adopt healthier routines [20].

Informal and Community-Based Health Education

Peer education, community talks, workshops: Kolawole et al. [21] evaluated the effectiveness of community-based health education programs in preventing NCDs. Their study found that such programs significantly improved participants’ knowledge, physical activity levels, dietary habits, and key health indicators like blood pressure, body mass index (BMI), and fasting glucose. The authors emphasized the importance of integrating culturally tailored messages, using digital tools, and involving local organizations to enhance impact. They recommended continued investment in these programs and further research to assess long-term outcomes and improve implementation strategies. Important next initiatives included addressing implementation issues, encouraging policy integration, and doing research to improve educational interventions. One important tactic for promoting global health equality and preventing NCDs is to continue funding health education programs. 

Religious, cultural, and social influencers: A cross-national survey demonstrated that perceptions of NCDs are significantly influenced by religious and cultural factors [22]. In Jordan, fatalistic beliefs rooted in religion often reduced an individual's sense of control over disease prevention, while in Tanzania, reliance on traditional healing and prevailing gender norms constrained women’s empowerment in health decisions. By contrast, participants in Peru expressed stronger confidence in lifestyle modification, those in the United States showed high awareness but inconsistent preventive action, and respondents in South Korea reflected a cultural emphasis on discipline and personal responsibility. These findings highlight that NCD prevention strategies must be context-sensitive, and integrate cultural and religious realities to be effective [22].

Odukoya et al. [23] highlighted the growing need for innovative and cost-effective strategies to address the rising prevalence of NCDs in low- and middle-income countries (LMICs). The authors noted that faith-based and faith-placed interventions can serve as effective avenues for health promotion and disease prevention in these contexts. Although empirical evidence remains limited, existing studies suggest that religious beliefs may influence behaviors that reduce the burden of NCDs, including lower tobacco and alcohol use. Moreover, the authors emphasize that in many emerging nations, political and religious goals can significantly shape health policies and individual health behaviors, underscoring the importance of considering cultural and spiritual factors when designing interventions [23].

A six-month intervention program in rural Northeast Brazil aimed to enhance the management of hypertension and diabetes through education and training [24]. Both before and after the procedures, measures of blood pressure and glycated hemoglobin (HbA1c) were made. The mean systolic and diastolic blood pressures of the hypertensive individuals under observation decreased by 13.4 and 5.8 mmHg, respectively. The HbA1c level decreased by 0.55% in individuals with diabetes. Patients with hypertension reached the ideal blood pressure level in 38.8% of cases, whereas patients with diabetes reached the intended HbA1c level in 16.9% of cases [24].

Health Learning Materials and Behavior Change Communication 

Information, Education, Communication (IEC) and Behavior Change Communication (BCC) tools: Governments play a crucial role in national health initiatives, particularly those aimed at changing health behaviors. IEC strategies, along with BCC, are essential for engaging communities and promoting sustainable behavioral changes. These approaches are applied in various settings, including schools, workplaces, and Anganwadi centers, targeting individuals from early childhood through adolescence. Together, IEC and BCC help foster community participation and support the adoption of healthy lifestyles [25]. In clinical settings and public health, BCC is essential for avoiding both communicable and noncommunicable illnesses. To encourage beneficial behaviors and maintain social, community, and individual improvements, it entails creating customized messages and strategies using a variety of communication channels. The study by Siswati et al. [26] investigates how tailored communication strategies can improve awareness and encourage positive lifestyle practices, such as nutrition, physical activity, and overall wellness. The research emphasizes the role of BCC in promoting health education and facilitating behavior change within communities, aiming to support healthier daily habits and long-term well-being, rather than addressing clinical or outbreak-related applications.

Visual aids, media campaigns, digital tools: A scoping review by Karan et al. [27] emphasizes the potential of digital education to help address the growing need for health professionals trained in the diagnosis, prevention, and management of NCDs. Digital learning has shown benefits for both pre-service and in-service health workers, helping to overcome geographic and logistical constraints and enhance workforce capacity. However, best practices for digital NCD education and lessons from global experiences remain underexplored. The authors also highlight challenges posed by the multilingual and diverse backgrounds of health professionals, which can complicate online learning. To overcome these challenges and optimize digital education strategies, they call for rigorous research, including assessments, quasi-experimental studies, and randomized controlled trials, supported by strong evidence and guidance from prior studies [27].

Effectiveness of health education in promoting healthy behaviors (e.g., physical activity, healthy eating, tobacco cessation): A meta-analysis conducted by Duan et al. [28] found that eHealth-based Multiple Health Behavior Change (MHBC) interventions such as physical activity, dietary habits, and weight management, significantly improved physical activity (Standardized Mean Difference or SMD=0.85, 95 % CI 0.23 to 1.47, p=0.008) and healthy dietary behaviors (SMD=0.78, 95 % CI 0.13 to 1.43, p=0.02) among individuals with NCD, compared with control groups; however, these interventions did not produce a significant effect on maintaining healthy weight (SMD=-0.13, 95 % CI -0.47 to 0.20, p=0.43). Subgroup analyses showed that theory-based interventions were more effective in promoting physical activity, whereas traditional delivery methods like SMS and phone calls were more effective in encouraging healthy dietary practices. The authors concluded that eHealth MHBC interventions hold promise for motivating patients with NCDs to adopt healthier behaviors, though further research is needed to refine intervention design and enhance effectiveness [28].

Barriers to effective health education in NCD prevention

Although health education is critical for preventing NCDs, a variety of barriers usually impede its effectiveness. People with limited health literacy struggle to understand risk factors, lifestyle modifications, and early warning signs of disease. Cultural concepts and inaccurate information exacerbate the problem, since conventions and preconceived assumptions may discourage people from seeking preventative care. The reach of health education projects is further hampered by a lack of funding and competent personnel, particularly in underserved or rural communities. As a result, irregular attempts fail to achieve long-term behavioral change. Despite these challenges, health education is an important method for supporting healthy lifestyles and preventing NCDs [29].

Language and accessibility barriers impede effective health education for NCD prevention. In multinational societies, communication can become unavailable due to a lack of resources in regional languages or culturally appropriate formats. People who live far away or have limited resources may find it difficult to access health education materials, exacerbating inequality and increasing susceptibility to chronic disease risk factors. Furthermore, limited political support and weak health-care systems may inhibit long-term progress. Governments often prioritize treatment over prevention, resulting in ineffective coordination, policy gaps, and poor monitoring of educational programs. In the absence of good governance, incorrect information and unhealthy lifestyle choices may spread faster than accurate health messages. To overcome these challenges, community-based initiatives, cultural adaptation, and continual education are required. Reducing the burden of NCDs necessitates improving healthcare systems and increasing political commitment [30].

Strategies and actions to improve health education for NCDs

Strategies and actions to improve health education for NCDs include several key approaches [31]. Early incorporation of preventive health subjects into school and university curricula aids in habit formation and cultivates lifelong knowledge of healthy living. Children frequently teach their parents skills, so prevention becomes ingrained in daily education and community culture, and this strategy also extends to families. Community health professionals play a vital role in education because they refute myths, offer factual and culturally relevant information, and encourage lifestyle modifications. Ongoing capacity building, oversight, and incentives increase their efficacy and ensure that communities always receive reliable guidance.

Persuasive messages may swiftly reach large populations through mass media channels, including social media, television, and radio. Mobile technology provides personalized reminders, advice, and instructional materials tailored to regional languages and literacy levels, effectively encouraging preventive behavior and sustaining change. Governments should support awareness efforts, regulate harmful industries, and incorporate programs into national policies to guarantee sustained health education. Collaboration with private firms, the media, and technology sectors can help build a supportive environment for NCD prevention by providing additional resources, innovation, and outreach [31].

Due to shifting patterns in health issues, approaches to maintaining and improving health have also evolved. Historically, efforts focused on treating illness with medicine and preventing disease through immunization. Today, however, medical advances alone are not enough. Despite scientific progress, many diseases remain without a cure, highlighting the importance of prevention in promoting better health outcomes. There are still major gaps in research on NCD education, especially in LMICs where few studies assess interventions over the long term. Mobile and digital health tools also remain underused. Success depends on equity and context-specific strategies, which guarantee the participation of marginalized groups and foster cooperation with community influencers and religious leaders. Global approaches differ from local contexts; for example, digital health programs are more feasible in high-income countries, while community-based strategies are often more effective in LMICs. Sustainability and long-term impact remain a gap in many studies, including evidence from Jordan, highlighting the need for follow-up and reinforcement. Future studies should prioritize equality and context-driven design while enhancing the long-term effects of education-based interventions, particularly in settings with low resources. They should also concentrate on digital learning, peer education, flexible models, and integrated curricula. 

Several intervention studies have evaluated the impact of health education on the prevention and management of non-communicable diseases across different settings. These studies vary in design, population, and outcomes, making a pooled statistical synthesis unfeasible. Therefore, a narrative synthesis approach was adopted. To enhance clarity and consistency, a structured summary of the included intervention studies, covering their design, setting, interventions, outcomes, and main findings, is presented in Table 1.

**Table 1:** Summary of some of the included studies on health education and NCDs NCDs: Non-communicable diseases; SBP: Systolic blood pressure; DBP: Diastolic blood pressure; HbA1c: Glycosylated hemoglobin; GP: General practitioners; SMD: Standardized Mean Difference; PRISMA: Preferred Reporting Items for Systematic Reviews and Meta-Analyses.

Author, Year	Country/Setting	Design & Sample size	Intervention	Outcomes measured	Key findings
Lemos Macedo et al., 2021 [24]	Brazil (rural)	Before-after study; hypertensive patients (n=276), diabetic patients (n=71)	Multicomponent community-based education and training	SBP, DBP, HbA1c	SBP ↓13.4 mmHg (p=0.021); DBP ↓5.8 mmHg (p<0.001); HbA1c ↓0.55% (p=0.185), but not significant. 38.8% reached target BP; 16.9% reached HbA1c goals; 38% had ≥1% HbA1c reduction.
Duan et al., 2021 [28]	Multi-country	Systematic review & meta-analysis of RCT; n=2,587 (total across 15 RCTs)	eHealth-based multiple health behavior interventions	Physical activity (PA), diet, and weight	PA: SMD=0.85 (95% CI 0.23-1.47, p=0.008); Diet: SMD=0.78 (95% CI 0.13-1.43, p=0.02); No significant effect on weight.
Almomani et al., 2021 [15]	Jordan (university students)	Pre–post study; n=178	3-week interactive educational sessions on NCDs	Knowledge, attitudes	Short-term improvements in knowledge and attitudes (p<0.05), but effects were not sustained at follow-up.
Claramita et al., 2021 [20]	Indonesia (high schools)	Quasi-experimental; peer-educator model; n=60 total: 10 GPs, 50 high school students	POSBINDU experiential peer-education program	Knowledge, perceptions, behaviors	Improved NCD knowledge and lifestyle perceptions among students and GPs; statistical details limited.
Kolawole et al., 2023 [21]	Nigeria (community)	Systematic review (PRISMA) of 73 studies	Culturally tailored health education programs	Knowledge, physical activity, diet, BMI, BP	Significant improvements in knowledge, PA, diet, BMI, and BP (p<0.05).

Limitations

Many studies had limited long-term follow-up, which restricted the assessment of sustained behavior change. Some relied on small sample sizes or case-study designs, limiting generalizability. Randomized controlled trials were relatively few, and potential publication bias existed due to the exclusion of grey literature. Heterogeneity in interventions, settings, and outcome measures also complicated comparisons across studies. These limitations should be considered when interpreting the findings and planning future research.

## Conclusions

Health education plays a central role in NCD prevention and control by reducing key risk factors, such as poor diet, inactivity, tobacco, and alcohol use, while simultaneously promoting positive behavior change and improving health literacy. Its effectiveness, however, is limited by barriers such as low literacy, cultural attitudes, a lack of resources, and insufficient health systems, emphasizing the importance of inclusive, context-sensitive interventions.

Health education remains among the most cost-effective and impactful strategies for reducing the global incidence of NCDs. It reduces healthcare expenses, promotes healthy lifestyles, and empowers individuals and communities. However, it requires multi-sectoral engagement, political commitment, and constant finance. Policymakers, business interests, education sectors, and health institutions should all collaborate to guarantee long-term viability, cultural sensitivity, and equitable access. With continuous innovation, health education will keep working to reduce NCD-related morbidity and mortality throughout the world.
